# Combining wood supply with reindeer foraging in the same forest: Evaluation of spacing and thinning strategies

**DOI:** 10.1007/s13280-025-02169-x

**Published:** 2025-04-19

**Authors:** Emma Holmström, Urban Nilsson

**Affiliations:** Southern Swedish Forest Research Centre, Swedish University of Agricultural Science, Box 190, 234 22 Alnarp, Lomma, Sweden

**Keywords:** Boreal, Forestry, Multi-use forestry, Plantation, Scots pine, Terricolous lichens

## Abstract

The forest land of northern Sweden is used for reindeer husbandry by the Indigenous Sámi while also being managed for wood supply. Modern forestry with dense pine *Pinus sylvestris* stands, maintained with high basal areas and high leaf areas, allow little light to reach the ground and the lichen cover. Finding sustainable forest management for low-productivity pine sites that combine lichen cover habitats with economically viable wood production is an urgent need. In this study, we compared pine regenerations resulting in 600, 1200 and 1800 trees per hectare when the stand reached a height of 10 m. In addition, we examined the effects of two thinning strategies: business as usual (BAU) follows thinning guidelines currently used in Swedish forests, whereas combined wood and lichen (CWL) features repeated heavy thinnings throughout the rotation. Results showed reduced production but a relatively small decline in economy in the CWL strategy compared to BAU, despite a large reduction in basal area. In addition, CWL resulted in larger but fewer trees per hectare which may also benefit biodiversity and the recreational use of the stands.

## Introduction

Boreal forests in Europe have a long continuous history of use for both reindeer herding and wood supply for pulp and timber. These two provisioning systems have different goals that determine the sustainability of their production. Crop production systems usually aim to optimize growth and yield based on soil and climate at a given site. Management is adapted to carrying capacity, and density-dependent measures underlie guidelines for spacing and basal area. Resilience in the system is coerced and anthropogenic (Rist et al. 2014; Felton et al. 2024).

In pastoral systems, like reindeer herding in boreal alpine and forest regions, mobility, diversity and flexibility are instead the fundamental strategies to ensure a steady resource supply and cope with limitations (Fernandez-Gimenez and Le Febre 2006). Reindeer herding pastoralists depend heavily on flexibility because of seasonal and annual variation in foraging conditions which require landscape-scale planning (Horstkotte et al. 2014).

A way forward for combined land use is to provide green infrastructure that enables mobility and flexibility, especially in the unpredictable era of climate change. Another important strategy is to provide a diversity of habitats within managed forests so that they become part of the green infrastructure and can provide suitable foraging habitat. Studies from both Sweden and Finland show a reduction in terricolous (forest floor) lichens in recent decades which correlates with changes in the forest structure (Kumpula et al. 2014; Sandström et al. 2016; Horstkotte and Moen 2019). Changing tree species (from Lodgepole pine to Scots pine) and adapting thinning and harvesting strategies might halt the current downward trend in lichen groundcover within 15 years (Eggers et al. 2023). Similarly, models based on Finnish national forest inventory data showed increase in ground cover lichens with heavy thinnings and/or prolonged rotation length (Miina et al. 2020). The experience from experiments with restoration of foraging habitats, lichen (Roturier et al. 2007, 2024) and peatland vegetation (Tarvainen et al. 2022) shows that although it might be technical and ecological possible, it is today costly and difficult.

Scots pine (*Pinus sylvestris*) is the most common tree species of Eastern Hemisphere boreal forests, especially on lower-fertility sites. It is important as a keystone species for the forested ecosystems, harbouring a range of field vegetation types, from berry-producing woody shrubs on more fertile sites to thick terricolous lichens on less-fertile and dry to dry-mesic sites. During the last century, forest researchers have established and measured (Nilsson et al. 2010) several long-term experiments on Scots pine stand management, providing data for growth and yield functions and decision support, considering spacing, competition and wood production (Pettersson 1992; Elfving and Kiviste 1997; Fahlvik et al. 2005; Nilsson et al. 2010; Ogana et al. 2022; Segtowich et al. 2023). A study with measurements of transplanted lichen growth in different habitats, mainly in Norway spruce dominated forests, showed increased growth with increasing site openness (Jonsson Čabrajič et al. 2010), and another study showed that ground lichen cover was reduced from abundant to moderate when forest density increased from 7 to 15 m^2^ in basal area (Horstkotte and Moen 2019). However, we found no experimental data on thinning Scots pine to maintain a continuously open canopy, providing the desired habitat for foraging. Therefore, in this study we have modelled the potential outcome of an intentional strategy to combine ecosystem services, using the empirical functions and data from our long-term experiments. We addressed the trade-offs between the currently deployed thinning strategy and an adapted strategy with suitable ground conditions for terricolous lichens, using a range of initial planting densities. We focused on three key outcomes: volume production, large-dimension trees and economic return on plantation investments. These indicators were compared for different rotation lengths before final harvest, using current legislation, production and profit as criteria for rotation length.

## Materials and methods

**Fig. 1:**
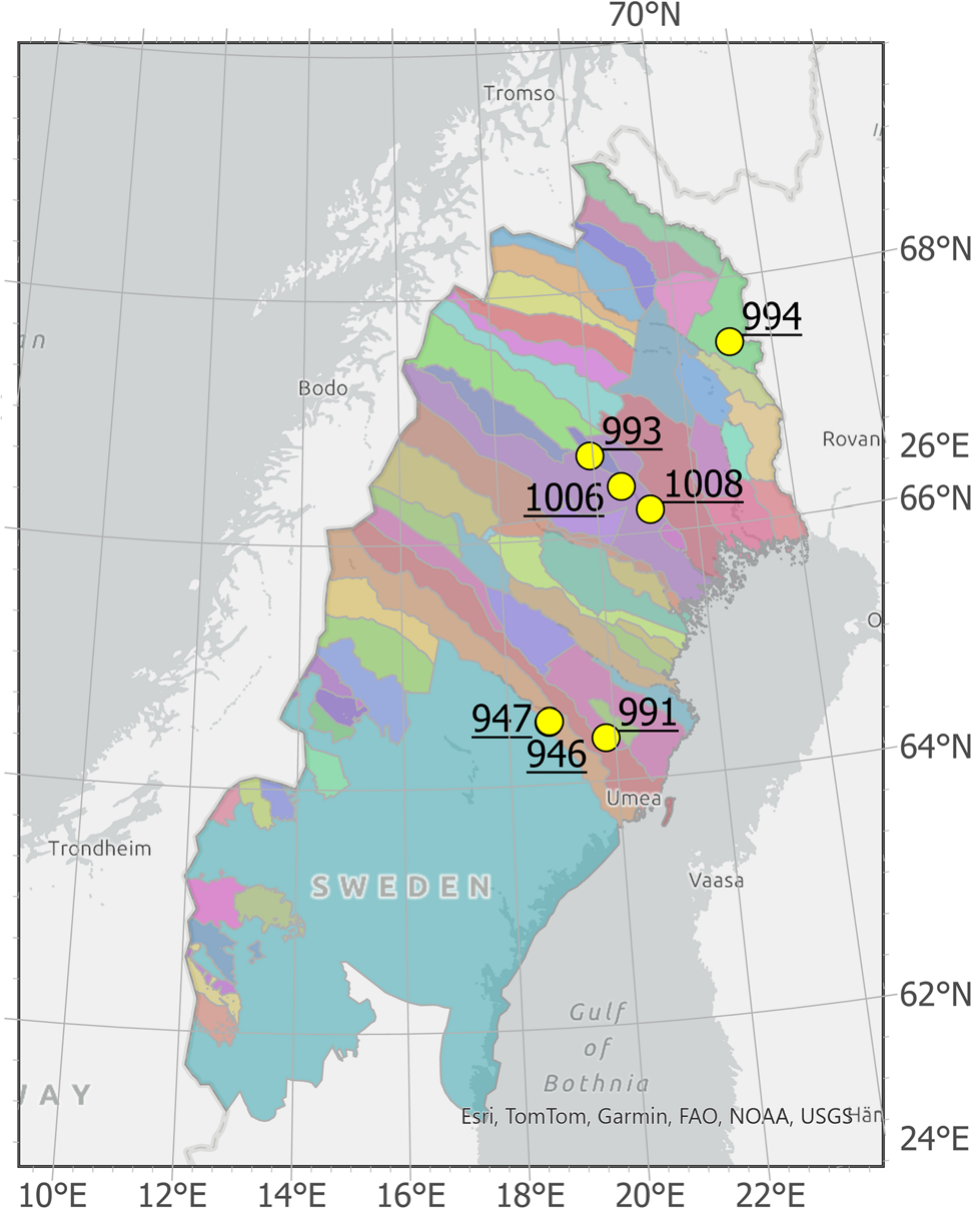
Map of the study area in northern Sweden. The different colours show the different reindeer herding communities. The yellow dots indicate the locations of the seven experimental sites. (Source: Sami Parliament)

This study generated stand development scenarios for pine forests with lichen-dominated understories, using actual site data from seven long-term experiments (Fig. 1),^1^, ^2^all from the same experimental series established between 1960 and 1980 (Nilsson et al. 2010). Based on site indicators and vegetation type, these were all low-fertility sites, with site indices (SIS) between 14 and 18 m top height at 100 years (Table 1). This corresponds to mean annual productions between 1 and 3 m^3^ ha^−1^ yr^−1^. The sites were selected based on their vegetation type which is focus of this study. We selected historically pine-dominated stands with field vegetation primarily of terricolous lichens, suitable for both wood production and reindeer foraging. However, the measured trees can also be used for productivity classifications using site index functions for pine (SIH), and for the seven sites, measurements have now been done for almost 100 years of total age. All of the sites increased productivity with SIH ranging between 19 and 26 m top height at 100 years, which corresponds to a mean annual production between 3 and 6 m^3^ ha^−1^ yr^−1^. In none of the seven sites, any ingrowth of pine was registered over the first hundred years.

**Table 1:** Initial values used in the Heureka simulations, derived from simulations of tree lists when top height reached 10 m with site characteristics of each experiment. Age = stand age (yr), SIS = site index on site indicators at the time of experiment establishment, SIH = site index from the latest measurement (total age 66–88 years), Dq = quadratic mean diameter (cm), BA = basal area (m^2^ ha^−1^), volume (m^3^ ha^−1^). Dq, basal area and volume were estimated with functions described below

Site	Age	SIS	SIH	600 stems ha^−1^			1200 stems ha^−1^			1800 stems ha^−1^		
				Dq	BA	Volume	Dq	BA	Volume	Dq	BA	Volume
946	28	18.4	26.3	16.6	12.2	60	13.8	16.4	80	12.5	19.6	95
947	31	15.9	24.6	16.4	11.9	59	13.7	16.0	78	12.4	19.1	92
991	32	16.6	24.1	16.2	11.6	57	13.5	15.6	76	12.2	18.6	89
993	44	17.3	19.6	15.6	10.7	52	13	14.5	69	11.8	17.2	82
994	45	14.8	19.3	15.6	10.7	52	13	14.4	69	11.8	17.2	82
1006	32	16.5	24.3	16.4	12.0	59	13.7	16.1	78	12.4	19.2	93
1008	32	16.7	24.2	16.3	11.9	58	13.6	15.9	77	12.4	19.0	92

Stands were simulated using three stem densities at 10 m top height (600, 1200 and 1800 trees ha^−1^) for all seven sites, for a total of 21 starting values. The development from planting to 10 m top height was simulated using a model developed by (Fahlvik et al. 2018) based on empirical data from long-term pre-commercial thinning experiments. The model outputs consist of a diameter distribution and parameters for the individual trees’ height-to-diameter relationships. Thereafter, the seven stands with model-derived tree lists of diameters and heights were imported into the Heureka decision support system StandWise (Fahlvik et al. 2014).

In Heureka, we simulated stand development based on two different thinning strategies: business as usual (BAU) and combined wood and lichen provisioning (CWL). In the BAU strategy, thinnings were done when basal area was around 25 m^2^ ha^−1^. The basal area was reduced by 30% in the first thinning and 25% in the second thinning. All thinnings were done as thinning from below with a thinning ratio (quadratic mean diameter of removed trees divided by quadratic mean diameter of retained trees) around 0.8–0.9. No thinning was done after the top height exceeded 21 m. For the CWL strategy, we created new thinning guidelines designed to ensure suitable habitat for terricolous lichens from first commercial thinning until final harvest. In CWL, the stand was thinned just before the basal area reached 20 m^2^ ha^−1^. A 40% removal of basal area left about 12 m^2^ ha^−1^ after thinning. Thinnings were done as thinning from below with thinning ratios around 0.8–0.9.

The simulated stem densities were assigned costs for regeneration, assuming 20% early seedling mortality and a cost of 4 SEK/seedling (10 SEK corresponding to ~ 1 €) in the ground which includes costs for seedlings, scarification and planting. Thinning operation costs followed Heureka standards. A price list for timber and pulpwood from northern Sweden was used, where the first logs were assigned to four quality classes from high (1) to low (4; Table 2). The first logs were distributed differently among these quality classes for the three spacings according to Fahlvik et al. (2018), where the sparse planting had no logs of the highest quality, supported by empirical data (Fahlvik et al. 2005).

**Table 2:** Percentages of first logs in different quality classes depending on number of planted seedlings in the different scenarios. Quality 1 is the best quality and quality 4 the worst

	1800 stems ha^−1^	1200 stems ha^−1^	600 stems ha^−1^
Quality 1	31	4	0
Quality 2	0	0	0
Quality 3	57	40	30
Quality 4	12	56	70

Heureka provides output for each five-year period of the simulation. For this study, we computed current annual increment (CAI), mean annual increment (MAI), stand age, quadratic mean diameter (Dq), stem density, top height, total volume production and net present value (NPV). Based on the Heureka output, the land expectation value (LEV) for each five-year-period was calculated, using a discount rate of 1.5%. Land expectation value (LEV) was calculated as:

$$\text{LEV}=\sum_{t=0}^{u}{a}_{t} {(1+r)}^{-t} \times \frac{{(1+r)}^{u}}{(({1+r)}^{u}-1)}$$

where a is net cost or income at time t, r is the discount rate, and u is rotation length (Faustman 1849/1995).

Similarly, average annual net revenues, here on called cash flow (CF), were calculated for each five-year-period (the sum of costs and revenue, without discount rate adjustment, divided by stand age). Timing of the final harvest was then evaluated based on either (i) the lowest legal age for final felling (LAFF) according to the Swedish Forest Act, or at the time of maximum value of, (ii) land expectation value, (iii) mean annual increment, (iv) cash flow. Simulations were done until all final harvest criteria were maximized for all stands which resulted in 135 years of total age.

## Results

**Fig. 2:**
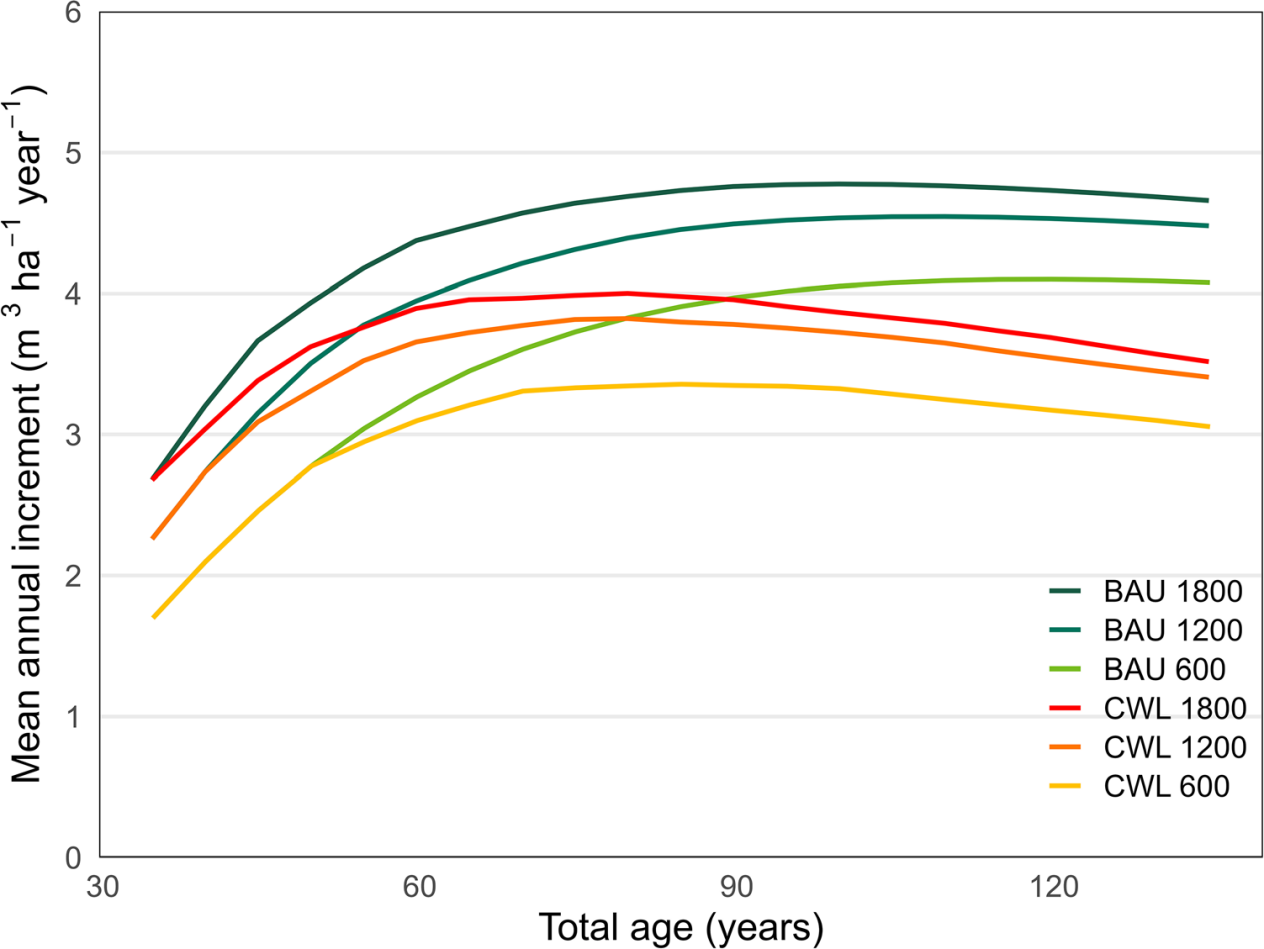
Development of mean annual increment (m^3^ ha^−1^ year^−1^) for the two thinning strategies (BAU, Business as usual; CLW, Combined wood and lichen provisioning) for different initial stem numbers at the top height of about 10 m (1800 stems ha^−1^, 1200 stems ha^−1^ and 600 stems ha^−1^). The figure shows mean values of the seven sites

As expected, higher planting densities resulted in higher stem densities at 10 m top height and had higher productivity at the beginning of the simulations. The business as usual (BAU) strategy provided a higher maximum mean annual increment than for the combined wood and lichen provisioning (CWL) strategy (Fig. 2). Maximum mean annual increment happened later for the BAU strategy than for CWL.

**Fig. 3:**
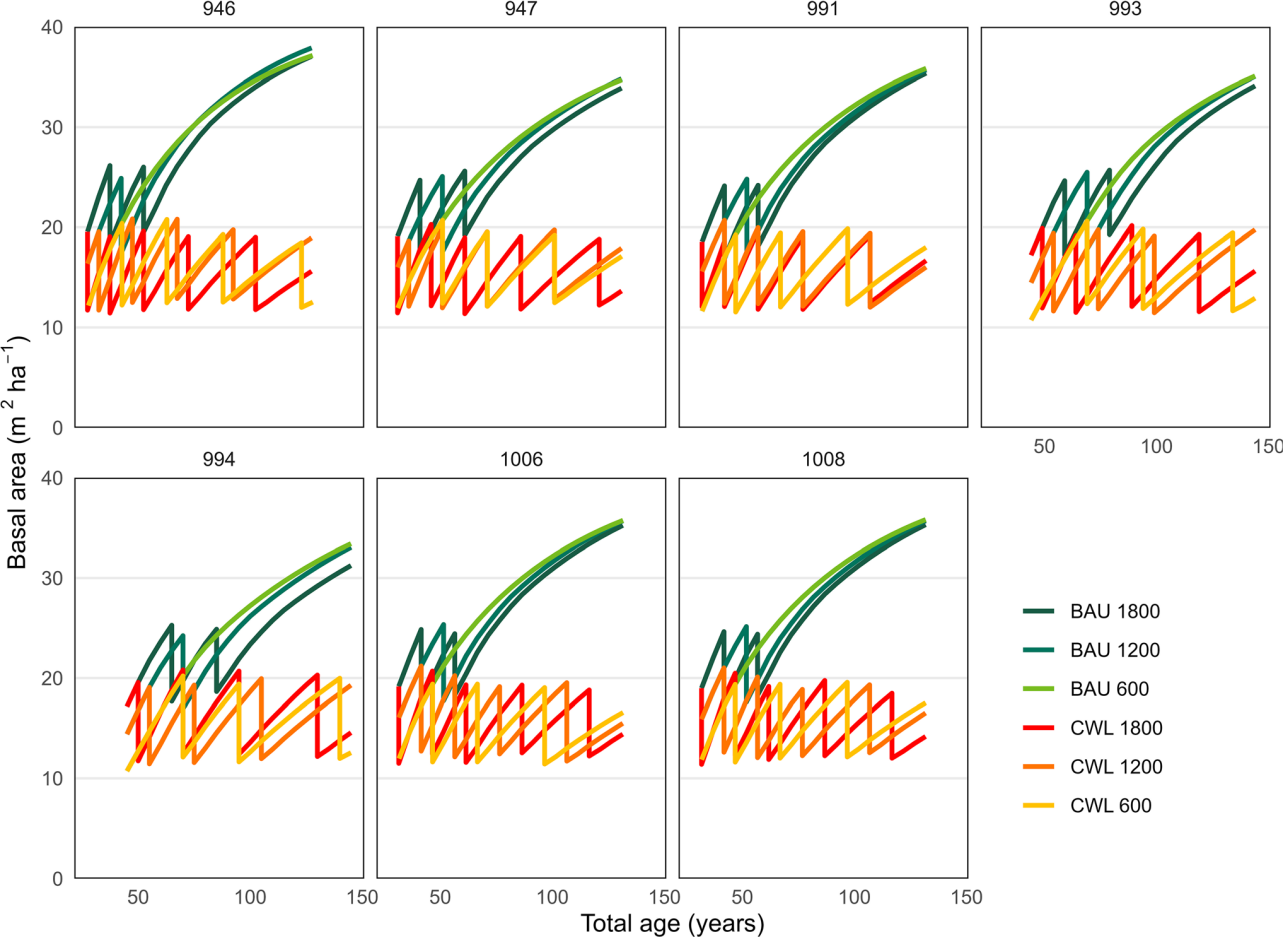
Basal area development for the seven sites, for the business as usual, (BAU) and combined wood and lichen management (CWL) scenarios at three different densities at 10 m top height over the simulated rotations

With the BAU strategy, all sites with the sparse planting density (600 seedlings ha^−1^) resulted in stand management without commercial thinning; the basal area never reached 25 m^2^ ha^−1^ before the top height reached 21 m (Fig. 3). All intermediate density stands (1200 st ha^−1^) sites were thinned once, and the densest stands (1800 st ha^−1^) were thinned twice.

With the CWL strategy, the sparse and intermediate stands were thinned 3–4 times, while the dense were thinned 4–5 times (Fig. 3).

**Fig. 4:**
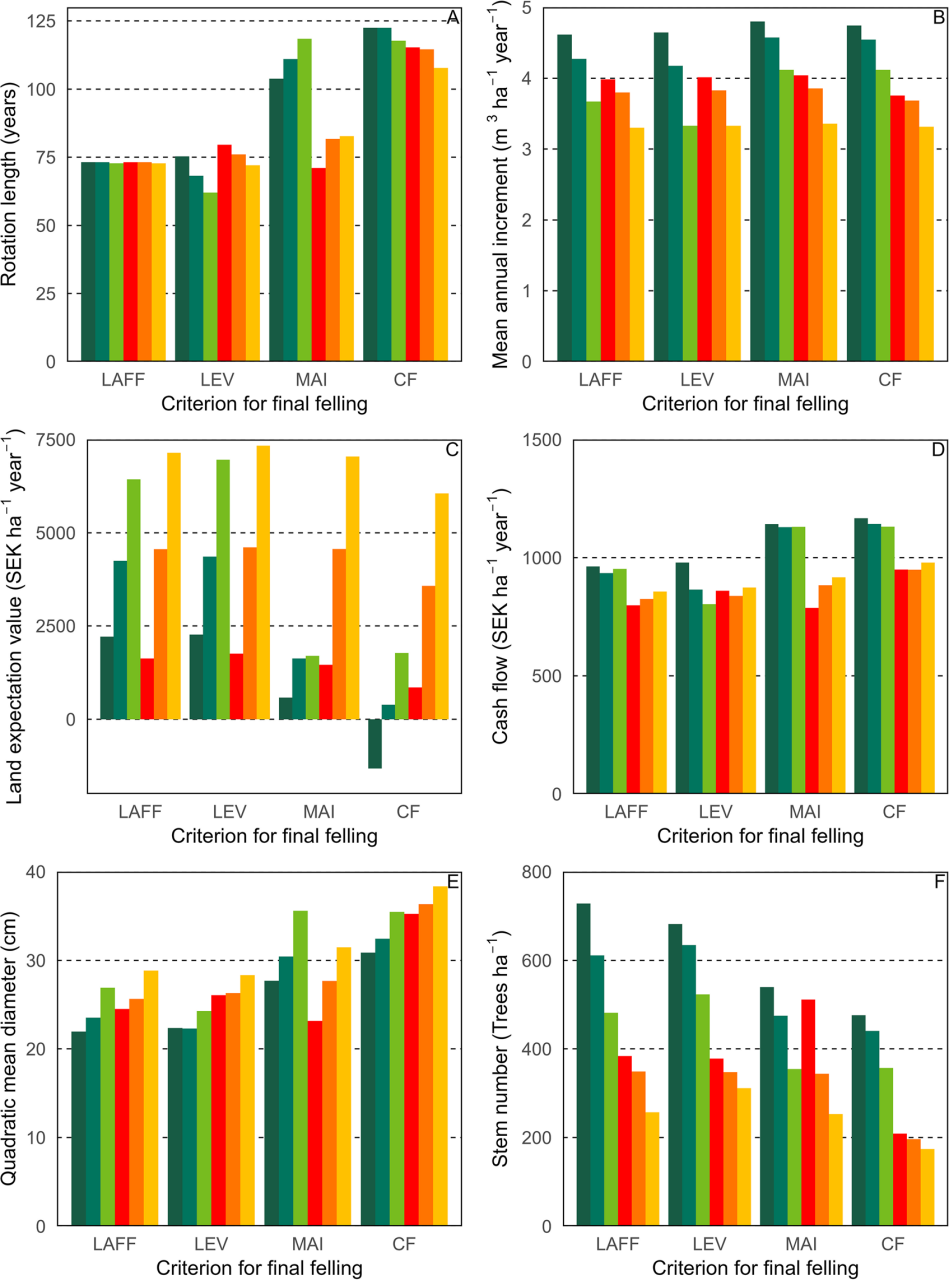
Simulation outcomes, as mean values of the seven sites, in terms of (A) rotation length (years), (B) mean annual increment (m^3^ ha^−1^ yr^−1^), (C) land expectation value at final felling (SEK ha^−1^ year^−1^), (D) cash flow (SEK ha^−1^ year^−1^), (E) quadratic mean diameter (cm) and (F) stem number at final felling (trees ha^−1^). The thinning scenarios business as usual (BAU; green colours) and combined wood and lichen provisioning (CWL; red colours). These are combined with three planting densities (1800—dark, 1200—intermediate, and 600—light seedlings ha^−1^). Rotation lengths were determined by four different criteria for final felling: lowest legal age of final felling (LAFF), maximum land expectation value (LEV), maximum mean annual increment (MAI) and maximum cash flow (CF)

Maximum cash flow resulted in the oldest final felling age (108–122 years). On average, rotation length was 45 years longer using cash flow as a criterion rather than land expectation values (Fig. 4). The shortest rotations varied among criteria, mainly depending on initial spacing. Using maximum land expectation value generated shorter rotations than the minimum legal age of final felling for sparse plantings (Fig. 4). Using maximum mean annual increment resulted in considerably longer rotations for CWL compared to BAU strategies. The varying results for profit and growth when comparing the different scenarios based on different criteria for final felling demonstrate that criterion for final felling will inevitably impact the result when comparing both the spacing and thinning strategies.

Volume production, expressed as mean annual increment at the time of final felling, was in all cases but one higher for BAU (range of means for seven sites 3.3–4.8 m^3^ ha^−1^ year^−1^) compared to CWL (range of means for seven sites 3.3–4.0 m^3^ ha^−1^ year^−1^) (Fig. 4).

The combination of a low stem density at planting (600 seedlings ha^−1^) and CWL resulted in 1.3–1.4 m^3^ ha^−1^ year^−1^ lower mean annual increment at final felling than densely planted seedlings (1800 seedlings ha^−1^) combined with BAU (Fig. 4). The combination of 1200 seedlings ha^−1^ with CWL resulted in 0.7–1.1 m^3^ ha^−1^ year^−1^ lower mean annual increment at final felling than the combination of 1800 seedlings ha^−1^ and BAU. The shorter rotations for the legal age of final felling and maximum land expectation value criteria only marginally reduced mean annual increment at final felling compared to the maximum mean annual increment criterion.

Financial return, in terms of land expectation value at final felling, increased with decreasing seedling planting density and was highest for 600 planted seedlings ha^−1^ and CWL (Fig. 4). CWL increased land expectation value at final felling over BAU for all combinations of planting densities and criteria for final felling except when dense planting (1800 seedlings ha^−1^) was combined with legal age of final felling or maximum land expectation value (Fig. 4). Longer rotations combined with BAU resulted in lower land expectation value at final felling for all scenarios.

Financial return in terms of cash flow was always higher for BAU than for CWL, except for sparse planting and short rotations.

Tree sizes, assessed by quadratic mean diameter, were higher for CWL than for BAU for all final felling criteria except maximum MAI. Given the same rotation length, quadratic mean diameter was always higher in CWL compared to BAU, but with maximum mean annual increment as the rotation length criteria, CWL always had a rotation at least 30 years shorter, which explains the result.

Stem density at the time of final felling was lower for CWL than for BAU for all criteria (Fig. 4) but stem density varied greatly among the different scenarios. When BAU was combined with dense planting and a short rotation, stem density was above 600 trees ha^−1^, compared to 200 stems ha^−1^ in CWL combined with long rotations (Fig. 4).

## Discussion

Lichen ground vegetation depends heavily on light reaching the forest floor (Jonsson Čabrajič et al. 2010), and measurements of lichen cover show a decrease, especially in younger stands which grow to become dense forests with higher basal area and canopy cover (Akujärvi et al. 2014; Horstkotte and Moen 2019). Therefore, we assumed that lichen production benefits from reducing the basal area by sparse planting and heavy thinning in Scots pine stands on low-fertility sites. The combination of the CWL strategy and sparse planting (600 stems ha^−1^) was the most extreme management regime to maintain lichen production. As expected, low seedling density at planting as well as heavy thinnings reduced production. However, during later stand development in a forest planted on a low-fertility site, the rate of change in mean annual increment is low. This means that production rates were relatively insensitive to the different rotation lengths in the simulations.

Low seedling density at planting has been shown to reduce production in several spacing experiments (Pettersson 1992; Nilsson and Albrektson 1994) but experiments with seedling densities as low as 600 seedlings ha^−1^ have, to our knowledge, never been reported for Scots pine. However, experimental reduction to 600 stems ha^−1^ using pre-commercial thinning resulted in significant production losses (Pettersson 1993). Nilsson et al. (2010) showed that a single heavy thinning to a basal area of 10 m^2^ ha^−1^ decreased stem wood production by 15% in Scots pine. Our simulated CWL stands were thinned heavily four to six times, but this only decreased MAI by 10–20% compared to the BAU scenario.

The main reason why land expectation value (LEV) increased with reduced planting density was a lower cost for seedlings and planting. This reduction comes early in the rotation and has a big effect on net present value and land expectation value. (Jonsson et al. 2022) also found that the financial return in terms of LEV was poor in plantations on low-fertility sites. When cash flow was used instead, there was little difference between the different planting densities. Lower costs at planting were offset by lower production and income at final felling for the lowest seedling density.

We did not expect that CWL would have higher land expectation value than BAU in many of the comparisons. The reason for this might be that the BAU scenario was developed for dense plantations, here represented by the 1800 seedlings ha^−1^ planting density. This had a higher LEV for BAU than CWL even though the differences were small. Thinning programmes for sparse plantations have been little studied, and restrictions in maximum top height for thinning in BAU resulted in no thinning of the sparse planting and only one thinning for the 1200 seedlings ha^−1^ planting density. Under CWL, the early income from thinnings increased LEV.

CWL generally decreased stem density and increased quadratic mean diameter compared to BAU. Low-density stands with a few large trees may be desirable from a recreational point of view, especially when combined with long rotations.

The criteria for final felling had a big influence on the rotation length. Rotations were generally shorter when lowest age of final felling and land expectation value were used as criteria, whereas cash flow resulted in longer rotations. Long rotations may increase carbon storage although at a greater damage risk (Pingoud et al. 2018). In these Heureka simulations, we used a mortality function to estimate average mortality, but it cannot capture stand replacing damage caused by wind or fire, so this is not part of the study framework.

The CWL thinning guidelines we designed for this study included thinnings in old and tall stands. After thinning, wind damage risk increases with the height of the stand (Valinger and Fridman 2011). Even if Scots pine is considered less sensitive than Norway spruce to wind damage (Felton et al. 2019), the risk is not negligible. This is one important reason why current guidelines do not recommend thinning in old stands with tall trees (Persson 1972).

In our study, we have focused on forest yield, in terms of volume, timber dimensions and revenue and made the assumption that a lower density would be beneficial for maintaining ground lichen coverage. This assumption is based on previous studies made specifically on lichens in the Northern European region (Jonsson Čabrajič et al. 2010; Horstkotte and Moen 2019; Miina et al. 2020). In a recent study by Eggers et al. 2023, the authors emphasized the importance of changing to an adapted management to prevent further losses of lichen cover in the northern boreal forests and the authors suggest to strategically focus on Scots pine stands with stand ages below 80 years. Also, the Finnish decision support simulation system, Motti, delivers the same response of higher lichen cover with strategies of sparse stands (heavy thinnings) or longer rotations (Miina 2020). In conclusion, this study shows that it can be difficult on these sites to adapt management to successfully provision lichens without losing production. However, on these low-fertility sites, mean annual increment in absolute values was relatively little affected by the CWL scenarios. In addition, land expectation value was higher for low-density planting followed by adapted thinnings and a combined wood- and lichen-adapted thinning guideline allowed trees to grow larger which may benefit other biodiversity indicators and the recreational value of the forest stands.

## Footnotes

1
https://sametinget.se/.

2
https://www.silvaboreal.com.
